# Discovery of Potential Orthosteric and Allosteric Antagonists of P2Y1R from Chinese Herbs by Molecular Simulation Methods

**DOI:** 10.1155/2016/4320201

**Published:** 2016-08-21

**Authors:** Xu Zhang, Fang Lu, Yan-kun Chen, Gang-gang Luo, Lu-di Jiang, Lian-sheng Qiao, Yan-ling Zhang, Yu-hong Xiang

**Affiliations:** ^1^Key Laboratory of TCM Foundation and New Drug Research, School of Chinese Material Medica, Beijing University of Chinese Medicine, Beijing 100102, China; ^2^Department of Chemistry, Capital Normal University, Beijing 100048, China

## Abstract

P2Y1 receptor (P2Y1R), which belongs to G protein-coupled receptors (GPCRs), is an important target in ADP-induced platelet aggregation. The crystal structure of P2Y1R has been solved recently, which revealed orthosteric and allosteric ligand-binding sites with the details of ligand-protein binding modes. And it suggests that P2Y1R antagonists, which recognize two distinct sites, could potentially provide an efficacious and safe antithrombotic profile. In present paper, 2D similarity search, pharmacophore based screening, and molecular docking were used to explore the potential natural P2Y1R antagonists. 2D similarity search was used to classify orthosteric and allosteric antagonists of P2Y1R. Based on the result, pharmacophore models were constructed and validated by the test set. Optimal models were selected to discover potential P2Y1R antagonists of orthosteric and allosteric sites from Traditional Chinese Medicine (TCM). And the hits were filtered by Lipinski's rule. Then molecular docking was used to refine the results of pharmacophore based screening and analyze the binding mode of the hits and P2Y1R. Finally, two orthosteric and one allosteric potential compounds were obtained, which might be used in future P2Y1R antagonists design. This work provides a reliable guide for discovering natural P2Y1R antagonists acting on two distinct sites from TCM.

## 1. Introduction

Human P2Y receptors are a family of nucleotide activated G protein-coupled receptors (GPCRs) which comprises eight subtypes [1]. According to the difference of coupling protein, eight subtypes are subdivided into two groups, named Gq-coupled P2Y1R and Gi-coupled P2Y12R. Gq-coupled P2Y1R (P2Y1, P2Y2, P2Y4, P2Y6, and P2Y11) can activate phospholipase C-*β* (PLC*β*) and promote intracellular calcium mobilization, while Gi-coupled P2Y12R (P2Y12, P2Y13, and P2Y14) can inhibit the adenylyl cyclase and activate certain ion channels [2]. Among the two groups, P2Y1R and P2Y12R play a significant role in ADP-induced platelet aggregation. The blockade of either receptor could effectively inhibit ADP-induced platelet aggregation and decreases thrombosis formation [3, 4]. Nowadays, antithrombotic drugs mainly act on P2Y12R but have some potential side effects, such as a long bleeding time and dyspnea [5–7]. Comparing with P2Y12R inhibitors, P2Y1R antagonists may offer an advantage of reducing bleeding liabilities [7]. Therefore, P2Y1R is becoming an especially attractive antithrombotic drug target. While still in the development stage, the antithrombotic drugs of P2Y1R antagonists are necessary for treatment thrombosis.

Consistent with all of the GPCRs, P2Y1R possesses the characteristics of the seven-transmembrane domain (7TMD). And it has two distinct sites, including orthosteric and allosteric site [8]. Orthosteric site located within the 7TMD, while allosteric site is situated at the lipidic interface of the 7TMD. Previous study [9] showed that orthosteric antagonists can completely block the ADP-mediated platelet aggregation and effectively reduce the formation of arterial thrombosis with only a slight prolongation of bleeding time. And allosteric antagonists [10] will accelerate the dissociation of its endogenous agonist 2MeSADP and substantially reduce platelet aggregation. It can also significantly control bleeding time with a limited effect on bleeding. Fortunately, the complex of the P2Y1R with orthosteric and allosteric antagonists was revealed, respectively, by Dr. Zhang et al. [8], which can help us understand the mechanisms of inhibiting ADP-induced platelet aggregation and provides a basis for discovering potential target compounds with less side effects and higher security. However, the crystal structure of P2Y1R with allosteric antagonists BPTU was discovered recently. Before then, some of compounds with similar biological activity as allosteric antagonists were regarded as orthosteric antagonists, which will have an adverse impact effect on the virtual screening based on ligand. Therefore, it is a primary problem to classify antagonists bound with orthosteric and allosteric site.

In this paper, 2D similarity search was used to distinguish antagonists of two distinct sites. Ligand-based similarity search is one of the common computed techniques to find similar structure of molecules based on 2D fingerprint [11]. The searching principle states that molecules with similar structures are likely to have similar biological activity. In this process, template molecule with certain biological activity was chosen as a query to search for similar molecules and the top-ranked molecules are likely to exhibit the required bioactivity [12–14]. Pharmacophore based screening and molecular docking would also be used in combination to discover potential P2Y1 antagonists acting on orthosteric and allosteric sites. Ligand-based pharmacophore models [15] can be established by extracting common chemical features that are responsible for their specific bioactivity, and the optimal model will be used to screen compounds which are arranged in the same relative orientation with the same features. Structure-based molecular docking is mainly employed to refine the results of pharmacophore based screening and analyze the interactions between proteins and small molecules.

Some constituents in Chinese herbal medicine have proven effectiveness in inhibiting ADP-induced platelet aggregation. For example, ginsenoside Rg2 exhibited stronger anticoagulation effects [16]. Therefore, it is possible to discover potential P2Y1 antagonists from Chinese herbs. The purpose of this study was to screen potential P2Y1 antagonists from Traditional Chinese Medicine Database (TCMD, version 2009) by using a series of molecular simulation methods. 2D similarity search was utilized to identify allosteric antagonists and orthosteric antagonists; Genetic Algorithm with Linear Assignment of Hypermolecular Alignment of Datasets (GALAHAD) was used to separately construct pharmacophore models of orthosteric and allosteric antagonists. Validated by built-in parameters and test set, two optimal pharmacophore models were selected as templates to search potential P2Y1 antagonists from the TCMD. Then molecular docking was utilized to refine the results of pharmacophore and analyze the receptor-ligand interactions. The results of virtual screening provide a basis for the discovery of potential antithrombotic lead compound acting on P2Y1R.

## 2. Materials and Methods

### 2.1. Pharmacophore Model Studies

#### 2.1.1. Classification of the Orthosteric and Allosteric Antagonists Acting on P2Y1R

Based on 2D fingerprints, similarity search can find compounds that are similar to the reference compound. Tanimoto coefficient [17] was used to measure the similarity by comparing the selected fingerprint property. Theoretically, the range of Tanimoto coefficient value is zero to one. The value is closer to one indicating the greater similarity between the two fingerprints of compound and reference compound.

By entering “human P2Y1 receptor antagonists” as a search term in the Binding Database (http://www.bindingdb.org/), 357 antagonists were obtained. MRS2500 and BPTU, two initial compounds which, respectively, bound with orthosteric and allosteric site, were selected as queries to search similar structures from 357 antagonists, respectively. All the parameters were set to the default value automatically.

Then 357 antagonists were successfully divided into two parts by 2D similarity search. The 2D similarity search results of orthosteric and allosteric compounds were listed in Tables 1 and 2, respectively. There is no industry standard for selecting the threshold of Tanimoto coefficient. For example, 0.3 and 0.5 were set as threshold in some literatures to identify compounds [18, 19]. From Tables 1 and 2, compounds with Tanimoto coefficient value higher than 0.7 can account for more than 90% of the total compounds. False-positive rate may be reduced by a relatively higher similarity threshold. Hence, 0.7 was selected as the threshold of Tanimoto coefficient to classify orthosteric and allosteric antagonists from all compounds. Then 55 orthosteric antagonists and 278 allosteric antagonists were obtained and there is no duplicated compound between the two parts. To insure the comparability of the data, 246 compounds with Ki value were selected from 278 allosteric antagonists. And those compounds will be employed to build train set and test set.

#### 2.1.2. GALAHAD Pharmacophore Hypotheses Generation

**Figure 1:**
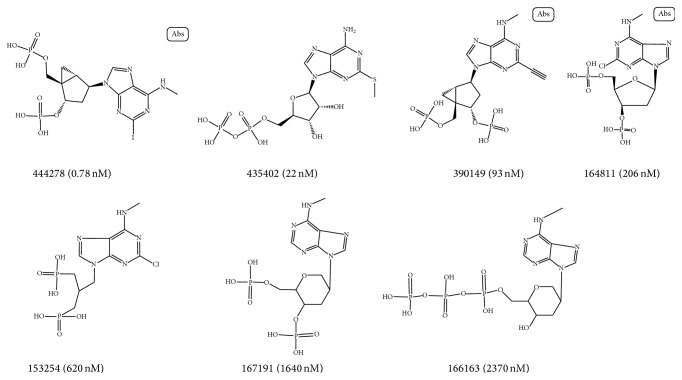
Structures, ID number, and the value of IC50 of 7 compounds in the training set for the generation of orthosteric pharmacophore hypothesis.

**Figure 2:**
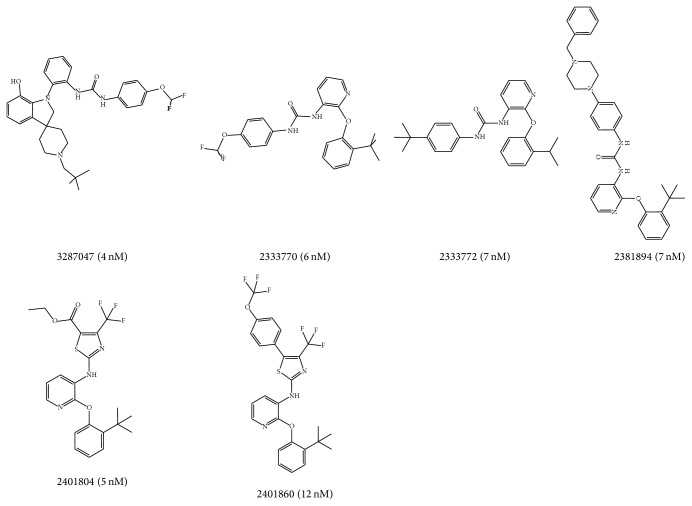
Structures, ID number, and the value of KI of 6 compounds in the training set for the generation of allosteric pharmacophore hypothesis.

The compounds which obtained by 2D similarity search had similar structures. Therefore, GALAHAD was selected as the pharmacophore modeling method, which is a typical modeling method based on compounds with similar structure. Based on the results of 2D similarity search, 7 P2Y1R orthosteric antagonists were selected to build orthosteric pharmacophore hypotheses. The remaining orthosteric antagonists were defined as active compounds of test set. Similarly, 6 P2Y1R allosteric antagonists were selected to generate allosteric pharmacophore hypotheses, and the remaining allosteric compounds were defined as active compounds of test set. The chemical information of the orthosteric compounds and allosteric compounds in the training sets was shown in Figures 1 and 2, respectively.

All the compounds were added into Tripos force field and then were minimized by Powell method with 1000 iterations. Then GALAHAD models were constructed based on the train set. Initially, the compounds in the training set aligned to each other in torsional space. And a genetic algorithm (GA) was operated to identify a set of compounds conformations with minimized strain energy and maximized pharmacophoric similarities. Then, the optimal set of conformations was aligned in Cartesian space as rigid-bodies. During the GA run, pharmacophore and steric quartets were employed to evaluate overlap. Twenty models were generated with different features, conformations, and overlay of the molecules [20, 21].

#### 2.1.3. Validation of the Pharmacophore Model

Internal parameters were calculated to evaluate the generated pharmacophore models firstly. The models were selected by three criteria [22]: (1) number of “hits” (*N*_Hits) should approximately equal the number of compounds which are used to generate the pharmacophore models; (2) the model needs to have lower Energy; (3) the models should have higher Specificity. The models which satisfied all the criteria would be selected and evaluated by the test set using UNITY module.

**Figure 3:**
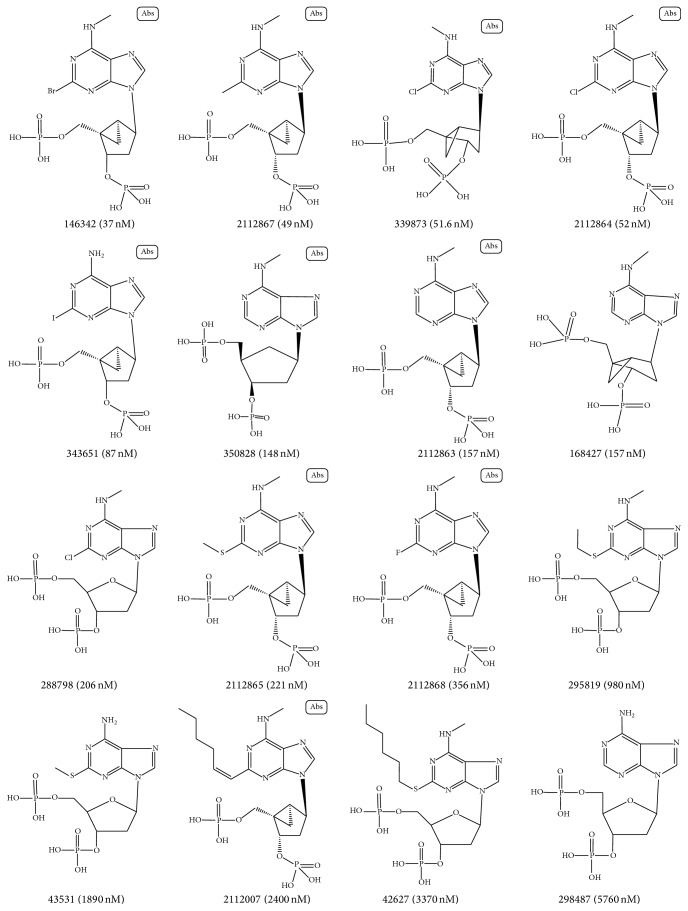
Structure, ID number, and biological values (IC50) of active compounds in the orthosteric test set.

**Figure 4:**
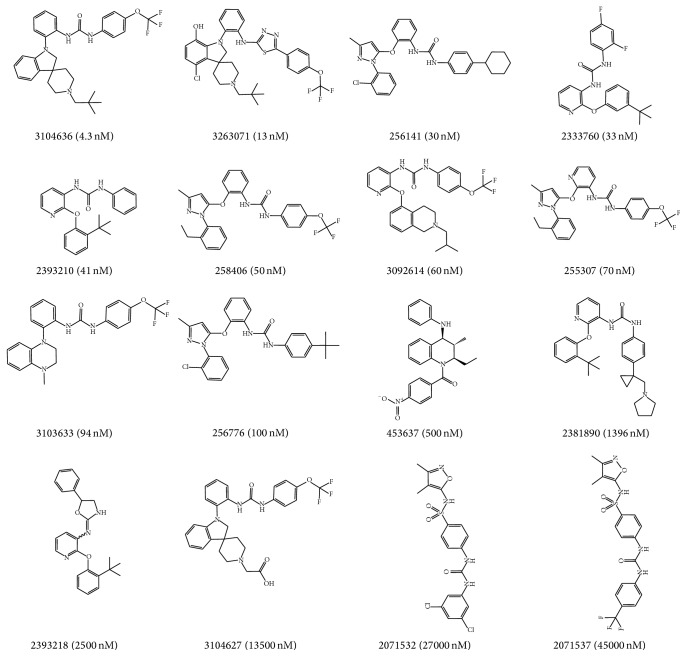
Structure, ID number, and biological values (KI) of active compounds in the allosteric test set.

One test set including 48 active compounds and 144 inactive compounds was used to evaluate the orthosteric pharmacophore models. And the other test set was used to evaluate the allosteric models which contained 240 active and 720 inactive compounds. Both of the inactive compounds were selected from the Binding Database. Part of the structures of compounds in the testing sets and the active values are shown in Figures 3 and 4.

The external parameters are presented as follows [23, 24]:* HRA* (the effectively hit ratio of active compounds),* IEI *(identified effective index), and* CAI* (comprehensive appraisal index). Considering all factors, the optimal pharmacophore model of orthosteric and allosteric antagonists was obtained, respectively.

### 2.2. Database Search

The optimal pharmacophore model of orthosteric and allosteric antagonists was utilized as queries to search the potential P2Y1R orthosteric and allosteric antagonists from TCMD. The “flexible database search” was carried out to perform the virtual screening process. Then, the hit compounds were filtered by Lipinski's rule of five (≥4), including MWT ≤ 500, A  Log *P* ≤ 5, H-bond donors ≤ 5, and H-bond acceptors ≤ 10 [25]. Compounds which meet the requirements were remained. Then, two lists of compounds including orthosteric and allosteric antagonists, with drug-like properties, were obtained. Finally, the two lists would be further analyzed in molecular docking study, respectively.

### 2.3. Molecular Docking Studies

#### 2.3.1. Define Binding Site

The crystal structures of the human P2Y1R-MRS2500 (PDB ID: 4XNW) and P2Y1R-BPTU (PDB ID: 4XNV) were obtained from the RCSB Protein Data Bank (http://www.rcsb.org/pdb/home/home.do). Common problems were automatically solved by Prepare Protein protocol, such as the lack of hydrogen, incomplete residues, the extra protein chains, and ligands. And the chain A of 4XNW and the whole chain of 4XNV were retained for docking. The binding pocket of P2Y1R orthosteric and allosteric antagonist was created, respectively, around the MRS2500 and BPTU using the Define and Edit Binding Site tools in Discovery Studio 4.0.

#### 2.3.2. Molecular Docking Strategy

LibDock and CDOCKER, two docking algorithms, were used to evaluate the applicability for the docking study of 4XNW and 4XNV. The initial compounds MRS2500 and BPTU were extracted from the active pockets and redocked into the corresponding crystal structure. By comparing the RMSD values between computed and experimental structures of initial compounds, the applicability of the two algorithms and the reasonability of the parameter settings were evaluated. In general, RMSD of less than 2.00 Å indicated that the docking algorithm could reproduce the binding mode of receptor-ligand. The RMSD is closer to zero, the better of docking results [26]. The docking algorithm with the smallest RMSD was selected for further employing.

In addition, in order to further validate the rationality of pharmacophore model and active pocket, the initial compounds were used to match the optimal model and analyze the interactions with active pocket of P2Y1R. And then compounds hit by two optimal pharmacophore models were docking into the crystal structure. Finally, potential P2Y1 orthosteric and allosteric antagonists which got higher docking score and formed favorable interaction with amino acid residue were obtained, respectively.

## 3. Results and Discussion

### 3.1. Pharmacophore Model Studies

#### 3.1.1. Pharmacophore Model Studies of Orthosteric Antagonist

Twenty models were produced by GALAHAD module based on a training set including seven active orthosteric compounds. Internal parameters such as *N*_Hits, Specificity, Energy, and Pareto Ranking were used to evaluate the models based on three criteria mentioned in Section 2.1.3. Firstly, ten models with values of “*N*_hits” more than 5 were displayed in Table 3, which indicated that at least six active compounds in the training set were mapped with each model. Secondly, Model O-06 and Model O-15 were discarded, because Energy of them is significantly higher than other models. Then a lower Specificity indicated that corresponding model did not have good discrimination. Model O-11, Model O-05, Model O-18, and Model O-16 with lower Specificity were also eliminated. Finally, the rest of four models were selected. Moreover, Pareto rank of these models was zero, which suggested that those models were not superior to each other.

**Figure 5:**
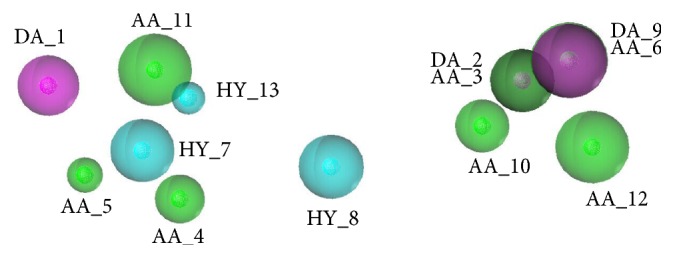
The optimal pharmacophore model (Model O-01) of orthosteric antagonist. Cyan indicates hydrophobic features, green indicates hydrogen bond acceptors, and purple indicates hydrogen bond donors.

The four pharmacophore models were validated by the test set of orthosteric antagonists; the results of validation were shown in Table 4. The* HRA* values of all those models were 100%, which indicated that all the models have the best ability to identify active compounds from test set. What is more, Model O-01 achieved the highest* IEI* and* CAI,* which indicated that Model O-01 had the best ability to identify active compounds from the inactive compounds. Thus, Model O-01 was chosen as the optimal pharmacophore model of orthosteric antagonists to screen the TCMD. The optimal model was shown in Figure 5 which included thirteen features: three hydrogen bond donors (DA_1, DA_2, and DA_9), seven hydrogen bond acceptors (AA_3, AA_4, AA_5, AA_6, AA_10, AA_11, and AA_12), and three hydrophobic features (HY_7, HY_8, and HY_13). Among them, DA_2 and AA_3 were generated in the same position, while AA_6 and DA_9 were also generated in the same position.

#### 3.1.2. Pharmacophore Model Studies of Allosteric Antagonist

Based on a training set including six allosteric antagonists, twenty GALAHAD models were derived. Similar to validation method of P2Y1R orthosteric antagonist, internal parameters were utilized to evaluate the models firstly. Six models were selected based on three criteria mentioned in Section 2.1.3, and then the models were validated by the test set of allosteric antagonist constructed previously.

The results of validated by test database were shown in Table 5. Model A-17 has highest value of* HRA*, which indicated that it has the strong ability to identify active compounds. However it has extremely low value of* IEI*, which suggests that it has poor ability to distinguish active compounds from inactive ones. Therefore, it cannot be regarded as the optimal model. The rest of five models with approximate values of* HRA* and the ability to identify active compounds are similar. However, Model A-07 has the highest value of* IEI* and* CAI*, which suggested that Model A-07 has the best ability to identify active compounds from the inactive compounds. Thus, Model A-07 was regarded as the optimal pharmacophore model of allosteric antagonists for screening the TCMD.

**Figure 6:**
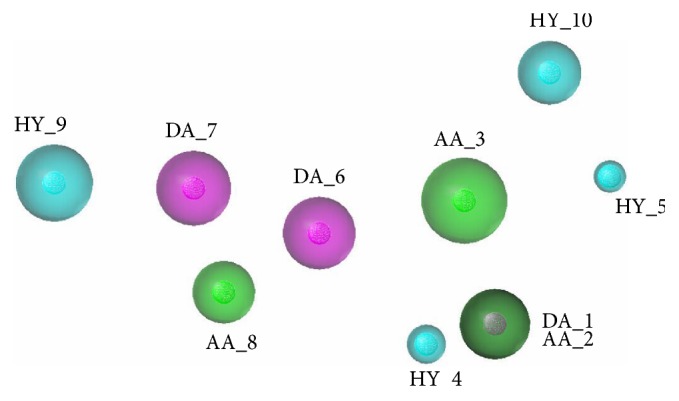
The optimal pharmacophore model (Model A-07) of allosteric antagonist. Cyan indicates hydrophobic features, green indicates hydrogen bond acceptors, and purple indicates hydrogen bond donors.

The optimal model was shown in Figure 6, which consisted of ten features, including three hydrogen bond donors (DA_1, DA_6, and DA_7), three hydrogen bond acceptors (AA_2, AA_3, and AA_8), and four hydrophobic features (HY_4, HY_5, HY_9, and HY_10). In Model A-07, a green sphere covered a purple sphere because DA_1 and AA_2 were generated in the same position.

### 3.2. Database Search

The Model O-01 of P2Y1R orthosteric antagonists and Model A-07 of P2Y1R allosteric antagonists were treated as the optimal pharmacophore models which were employed to screen P2Y1R antagonists from TCMD, respectively. Then, two lists were obtained including 3480 and 6229 compounds, respectively. During this process, the QFIT value was computed to assess the matching degree between compounds and pharmacophore models, and a higher value of QFIT indicated that corresponding compound was mapped well with the pharmacophore model. Then further filtering based on “Lipinski's rule of five” was implemented; 798 and 961 potential drug-like P2Y1R orthosteric antagonists and allosteric antagonists were obtained, respectively. Finally, the antagonists would be docked into corresponding active pockets of P2Y1R, respectively, by using molecular docking algorithm.

### 3.3. Molecular Docking Studies

#### 3.3.1. Orthosteric Antagonists Molecular Docking

The orthosteric active pocket was created with a sphere radius of 9.50 Å around the initial compound MRS2500 in 4XNW. Then, the MRS2500 was redocked into the crystal structure by using LibDock and CDOCKER, respectively, and the RMSD values of corresponding two docking algorithms were 3.81 Å and 0.58 Å. Therefore, CDOCKER with the value of RMSD less than 2.00 Å was chosen for docking study of orthosteric antagonists based on the smaller RMSD.

**Figure 7:**
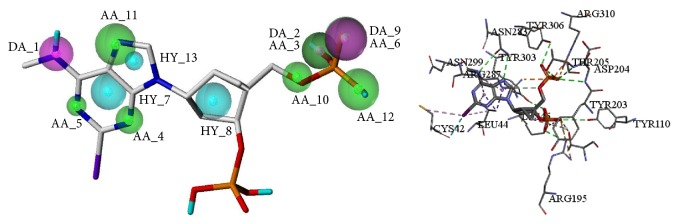
Pharmacophore mapping results and molecular docking results of MRS2500.

In addition, the initial compound, MRS2500, was used to match the optimal pharmacophore model (Model O-01) and analyze the interactions with orthosteric active pocket of P2Y1R. The mapping results of MRS2500 with Model O-01 and the interactions results between MRS2500 and amino acid residue were shown in Figure 7. The initial compound MRS2500 was mapped well with all the features of pharmacophore model. The purine ring which mapped features of HY_7 and HY_13 also formed hydrophobic interactions with LEU44 and ARG287. And the phosphate group formed hydrogen bond interactions with TYR306, THR205, and ASP204, which were also mapped with features of DA_2, AA_3, and AA_12, respectively. Thus, the analysis results of pharmacophore modeling and molecular docking were almost consistent which suggested that the study is reliably.

Then, 798 drug-like compounds which were filtered by the optimal pharmacophore model were docked into the orthosteric active pocket, resulting in a hit list of 285 compounds. The compounds docking score was expressed by -CDOCKER_ENERGY, and a high -CDOCKER_ENERGY corresponds to a favorable binding mode between compound and P2Y1R. And the compounds, which had higher docking score and QFIT, were selected as potential compounds for further research. Finally, among the potential compounds, (2R)-hydroxy-4-(9-adenyl)butyric acid and ganoderpurine were regarded as potential P2Y1R orthosteric antagonists. The two candidates have got higher -CDOCKER_ENERGY score, QFIT value, and similar interaction modes with P2Y1R-MRS2500. In addition,* Lentinus edodes*, the source herbal medicine of (2R)-hydroxy-4-(9-adenyl)butyric acid, proved to be effective in inhibiting the aggregation of platelets [27].

**Figure 8:**
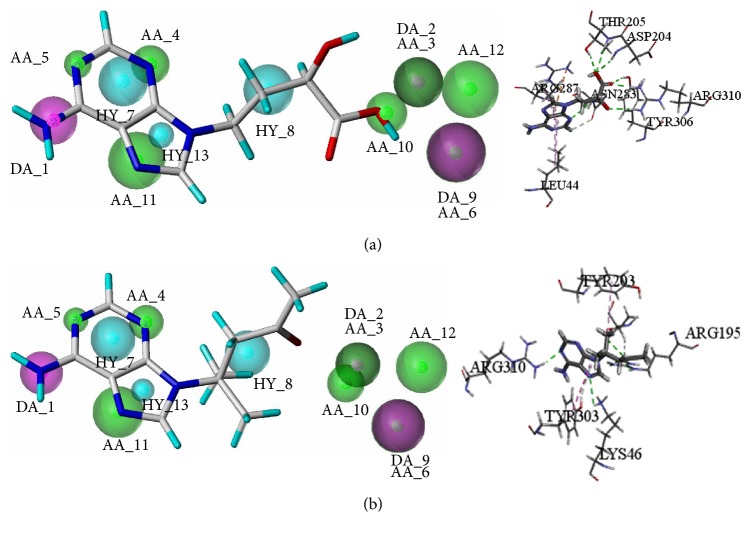
Pharmacophore mapping results and molecular docking results of (2R)-hydroxy-4-(9-adenyl)butyric acid (a) and ganoderpurine (b).

More specifically, (2R)-hydroxy-4-(9-adenyl)butyric acid got a higher QFIT 81.24 and was mapped with eight features of the optimal pharmacophore model. The compound got a higher -CDOCKER_ENERGY score 30.96 and formed hydrogen bond interactions with ASP204, THR205, ASN283, and TYR306 and also formed hydrophobic interactions with LEU44 and ARG287. What is more, when the purine ring of the compound was mapped with features of HY_7 and HY_13, the amino acids residues LEU44 and ARG287 also formed hydrogen bond interactions with the compounds. In terms of hydrophobic interactions, the nitrogen atom formed hydrogen bonds with TYR303 and was also mapped with features of AA_4 (Figure 8(a)).

Ganoderpurine well mapped seven features with the Model O-01 and the QFIT value was 90.19. Besides, ganoderpurine achieved a higher -CDOCKER_ENERGY score 26.48. It also formed hydrogen bond interactions with TYR303 and formed hydrophobic interactions with TYR203. Furthermore, the atoms which formed hydrogen bonds and hydrophobic interactions with TYR303 and TYR203 were also mapped with AA_4 and HY_8, respectively (Figure 8(b)). Therefore, the result of pharmacophore modeling study agrees with the molecular docking study, which suggested that the above results are credible.

Besides, two compounds, eckol and nodakenin which can inhibit the aggregations of platelets [28, 29], were hit by present study. And the source herbal medicines of the other four compounds have been found pharmacological effects of inhibiting the aggregation of platelets [27, 30–32]. The four compounds and source herbal medicines were as follows: icariol A1 (*Epimedium sagittatum*), lentysine (*Lentinus edodes*), boeravinone E (*Boerhavia diffusa*), and 6-demethoxycapillarisin (*Artemisia capillaris*-derived). To some extent, this further proved the reliability of the results of present study.

#### 3.3.2. Allosteric Antagonists Molecular Docking

Similarly, the initial compound BPTU was employed to define the active pocket of allosteric site of P2Y1R. And the radius of the binding pocket was 10.16 Å. The RMSD was calculated after the initial compound BPTU was redocked into the crystal structure using LibDock and CDOCKER, respectively, and the corresponding RMSD values were 0.74 Å and 0.61 Å. In that case, both docking algorithms could reproduce the P2Y1-BPTU binding model reasonably. However, the closer the RMSD is to zero, the more reliable the results of the corresponding docking procedure is. Thus, CDOCKER was selected for further docking study of allosteric antagonists.

**Figure 9:**
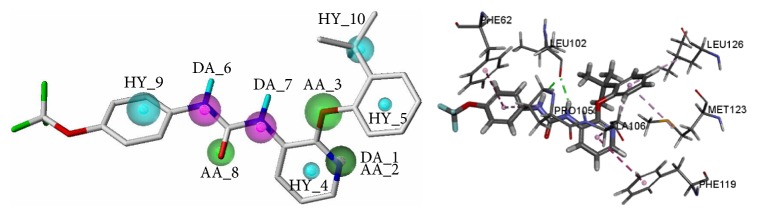
Pharmacophore mapping results and molecular docking results of BPTU.

To contrast result of pharmacophore model and docking, the initial compound of allosteric site, BPTU, was used to match the optimal pharmacophore model (Model A-07) and analyze the interactions with allosteric active pocket of P2Y1R. The mapping results of BPTU with Model A-07 and the interactions results between BPTU and allosteric binding site were shown in Figure 9. From Figure 9, it found that the initial compound BPTU was mapped well with all the features of the Model A-07. Hydrogen bonding and hydrophobic interactions were the major interactions between ligand and receptor. To be specific, BPTU formed hydrogen bond interactions with LEU102 and hydrophobic interactions with PHE62, PRO105, LEU126, and MET123, which was consistent with literature [8]. What is more, pyridyl group mapped with HY_4 also formed hydrophobic interactions with ALA106 and PHE119, while the benzene rings which formed hydrophobic interactions with LEU126, MET123, and PHE62 were also mapped with the corresponding hydrophobic features. In conclusion, the analysis results of pharmacophore modeling and molecular docking were almost consistent, and this indicated the rationality of the result.

**Figure 10:**
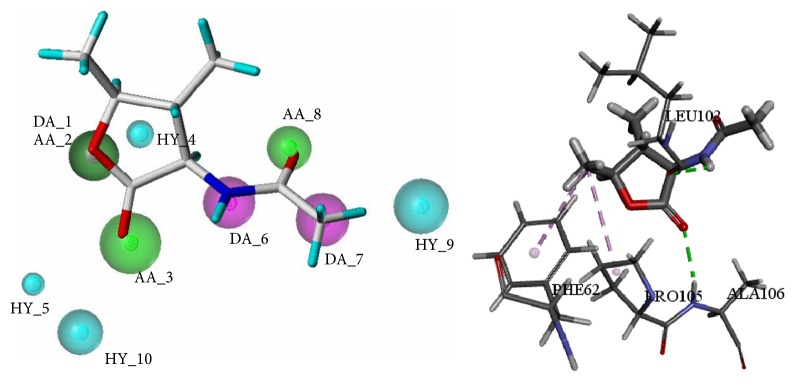
The results of with desmodilactone mapping and molecular docking.

In the following study, 961 drug-like compounds obtained from the result of pharmacophore model were docked into the allosteric active pocket. Similar to orthosteric antagonists, the docking score was expressed by -CDOCKER_ENERGY. Taking value of -CDOCKER_ENERGY and QFIT into consideration, compounds with high-scoring function values were retained. And these compounds were treated as potential P2Y1R allosteric antagonists. Finally, among this potential compounds, desmodilactone was regarded as most promising P2Y1R allosteric antagonist. For instance, desmodilactone was mapped with seven features of Model A-07. And the QFIT value and -CDOCKER_ENERGY score were 50.91 and 15.98, respectively. Then from the analysis of the key amino acids, desmodilactone formed hydrogen bond interactions with LEU102 and formed hydrophobic interactions with PHE62 and PRO105. Furthermore, the amino acids residue LEU102 formed hydrogen bonds with the compound, and the oxygen atom of the compound has also formed a hydrogen interaction with the same amino acid (Figure 10). Moreover, the result of molecular docking and pharmacophore was almost uniform, which indicated that the above results are reasonable.

Previous studies [33] showed that rhinacanthin Q hit by virtual screening has the effect on inhibiting the platelet aggregation. In addition, herbal medicines of other four potential compounds were usually used to treat Blood Stasis Syndrome based on Chinese medicine theory. The compounds and herbal medicines were as follows: 6-methylgingediol (*Zingiber officinale*), dehydrohirsutanonol (*Viscum cruciatum*), 5-O-caffeoyl quinic acid butyl ester (*Erigeron breviscapus*), and Delamide (*Aconitum carmichaeli*). Furthermore, the former two herbal medicines,* Zingiber officinale* [34] and dehydrohirsutanonol have proven [35] to be the effective Chinese medicine in inhibiting the aggregation of platelets. On the basis of the above analysis, the results of pharmacophore and molecular docking are reliable.

## 4. Conclusions

In recent years, thrombosis has been a serious threat to human health. P2Y1R antagonists, whether recognizing orthosteric or allosteric sites, have a unique advantage in clinical safety of medication. In the paper, series technologies of computer aided drug design were jointly used, including 2D similarity search, pharmacophore modeling, and molecule docking. 2D similarity search firstly attempted to solve the problem of inappropriate classification of P2Y1R antagonists. Then, 55 orthosteric antagonists and 246 allosteric antagonists were obtained. In the following study, GALAHAD models of orthosteric and allosteric antagonists of P2Y1R were generated separately. And Model O-01 and Model A-07 were treated as the optimal pharmacophore model, respectively, to retrieve potential compounds from TCMD. Finally, crystal structures of P2Y1R were used to refine the hit compounds and analyze the ligand interaction mode between hits and P2Y1R.

In conclusion, the study successfully attempted to use the crystal structure of P2Y1R and series of computational approaches to discovery of potential P2Y1R antagonists bound with two distinct sites from Chinese herbs. Finally, three potential P2Y1R antagonist leads were obtained. To be specific, (2R)-hydroxy-4-(9-adenyl)butyric acid and ganoderpurine were regarded as promising P2Y1R orthosteric antagonists, while desmodilactone was treated as the most potential allosteric P2Y1R antagonist. The result was expected to be further employed in the search for potential natural P2Y1R orthosteric and allosteric antagonist. The shortcoming of this study was without combining the biological experiments. In the future, molecular simulation methods and biological experiments will be combination used to discover potential novel antithrombotic drugs.

## Figures and Tables

**Table 1:** Similarity search results of orthosteric antagonists.

Tanimoto coefficient^a^	Number^b^	Percent^c^
0 < *T* ≤ 0.5	0	0%
0.5 < *T* ≤ 0.6	2	3.45%
0.6 < *T* ≤ 0.7	1	1.72%
0.7 < *T* ≤ 0.8	28	48.28%
0.8 < *T* ≤ 0.9	12	20.69%
0.9 < *T* ≤ 1.0	15	25.86%

*Total*	58	100.00%

^a^Tanimoto coefficient is the index of similarity.

^b^Number is the number of compounds within in the corresponding threshold value of Tanimoto coefficient.

^c^Percent is percentage of the number of compounds.

**Table 2:** Similarity search results of allosteric antagonists.

Tanimoto coefficient^a^	Number^b^	Percent^c^
0 < *T* ≤ 0.5	6	2.01%
0.5 < *T* ≤ 0.6	6	2.01%
0.6 < *T* ≤ 0.7	9	3.01%
0.7 < *T* ≤ 0.8	83	27.76%
0.8 < *T* ≤ 0.9	173	57.86%
0.9 < *T* ≤ 1.0	22	7.36%

*Total*	299	100.00%

^a^Tanimoto coefficient is the index of similarity.

^b^Number is the number of compounds within in the corresponding threshold value of Tanimoto coefficient.

^c^Percent is percentage of the number of compounds.

**Table 3:** Twelve GALAHAD models selected based on *N*_Hits.

*N*_Hits Model	*N*_Hits	Specificity	Energy	Pareto
O-12	7	2.37	41.35	0
O-06	7	1.95	22860.18	0
O-11	6	0.84	0.80	0
O-05	6	1.82	−0.30	0
O-20	6	2.18	0.12	0
O-18	6	1.77	−0.52	0
O-16	6	1.44	0.45	0
O-14	6	2.50	−0.05	0
O-15	6	3.72	2134232.75	0
O-01	6	2.30	12.39	0

**Table 4:** The validation results of selected six GALAHAD models.

Model	*N*_Hits	Specificity	Energy	Pareto	Ha^a^	Ht^b^	*HRA* ^c^	*IEI* ^d^	*CAI* ^e^
*O-01*	*6*	*2.30*	*12.39*	*0*	*48*	*56*	*100.00%*	*3.429*	*3.429*
O-12	7	2.37	41.35	0	48	164	100.00%	1.171	1.171
O-20	6	2.18	0.12	0	48	64	100.00%	3.000	3.000
O-14	6	2.50	−0.05	0	48	91	100.00%	2.110	2.110

^a^Ha is the number of active compounds screened by a pharmacophore model.

^b^Ht is the total number of compounds screened by a pharmacophore model.

^c^ *HRA* represents the ability to identify active compounds from the test set.

^d^ *IEI* represents the ability to identify active compounds from nonactive compounds.

^e^ *CAI* is the comprehensive appraisal index.

**Table 5:** The validation results of allosteric pharmacophore models.

Model	*N*_Hits	Specificity	Energy	Pareto	Ha^a^	Ht^b^	*HRA* ^c^	*IEI* ^d^	*CAI* ^e^
*A-07*	*6*	*3.31*	*34.02*	*0*	*214*	*358*	*89.17%*	*2.39*	*2.132*
A-01	6	3.44	15.65	0	220	486	91.67%	1.81	1.660
A-03	6	3.44	37.63	0	218	375	90.83%	2.33	2.112
A-04	6	3.44	37.63	0	218	375	90.83%	2.325	2.112
A-13	6	3.44	38.09	0	216	449	90.00%	1.924	1.732
A-17	6	2.68	14.82	0	234	842	97.50%	1.112	1.084

^a^Ha is the number of active compounds screened by a pharmacophore model.

^b^Ht is the total number of compounds screened by a pharmacophore model.

^c^ *HRA* represents the ability to identify active compounds from the test set.

^d^ *IEI* represents the ability to identify active compounds from nonactive compounds.

^e^ *CAI* is the comprehensive appraisal index.

## References

[1] Costanzi S., Mamedova L., Gao Z.-G., Jacobson K. A. (2004). Architecture of P2Y nucleotide receptors: Structural comparison based on sequence analysis, mutagenesis, and homology modeling. *Journal of Medicinal Chemistry*.

[2] Jacobson K. A., Ivanov A. A., Castro S. D., Harden T. K., Ko H. (2009). Development of selective agonists and antagonists of P2Y receptors. *Purinergic Signalling*.

[3] Pfefferkorn J. A., Choi C., Winters T. (2008). P2Y1 receptor antagonists as novel antithrombotic agents. *Bioorganic and Medicinal Chemistry Letters*.

[4] Costanzi S., Tikhonova I. G., Ohno M. (2007). P2Y_1_ antagonists: combining receptor-based modeling and QSAR for a quantitative prediction of the biological activity based on consensus scoring. *Journal of Medicinal Chemistry*.

[5] Wijeyeratne Y. D., Heptinstall S. (2011). Anti-platelet therapy: ADP receptor antagonists. *British Journal of Clinical Pharmacology*.

[6] John J., Koshy S. K. G. (2012). Current oral antiplatelets: focus update on prasugrel. *Journal of the American Board of Family Medicine*.

[7] Gachet C. (2008). P2 receptors, platelet function and pharmacological implications. *Thrombosis & Haemostasis*.

[8] Zhang D., Gao Z.-G., Zhang K. (2015). Two disparate ligand-binding sites in the human P2Y_1_ receptor. *Nature*.

[9] Hechler B., Nonne C., Eun J. R. (2006). MRS2500 [2-iodo-*N*
^6^-methyl-(*N*)-methanocarba-2′- deoxyadenosine-3′,5′-bisphosphate], a potent, selective, and stable antagonist of the platelet P2y1 receptor with strong antithrombotic activity in mice. *Journal of Pharmacology and Experimental Therapeutics*.

[10] Chao H., Turdi H., Herpin T. F. (2013). Discovery of 2-(phenoxypyridine)-3-phenylureas as small molecule P2Y_1_ antagonists. *Journal of Medicinal Chemistry*.

[11] Willett P. (2009). Similarity methods in chemoinformatics. *Annual Review of Information Science and Technology*.

[12] Johnson M. A., Maggiora G. M. (1990). Concepts and applications of molecular similarity. *American Mathematical Monthly*.

[13] Sheridan R. P. (2007). Chemical similarity searches: when is complexity justified?. *Expert Opinion on Drug Discovery*.

[14] Stumpfe D., Bajorath J. (2011). Similarity searching. *Wiley Interdisciplinary Reviews: Computational Molecular Science*.

[15] Yang S.-Y. (2010). Pharmacophore modeling and applications in drug discovery: challenges and recent advances. *Drug Discovery Today*.

[16] Li C. T., Wang H. B., Xu B. J. (2013). A comparative study on anticoagulant activities of three Chinese herbal medicines from the genus Panax and anticoagulant activities of ginsenosides Rg1 and Rg2. *Pharmaceutical Biology*.

[17] Willett P. (2006). Similarity-based virtual screening using 2D fingerprints. *Drug Discovery Today*.

[18] Yao S., Lu T., Zhou Z. (2014). An efficient multistep ligand-based virtual screening approach for GPR40 agonists. *Molecular Diversity*.

[19] Reddy S. V. G., Reddy K. T., Kumari V. V. J. (2014). Ligand based virtual screening to identify potential anti cancer ligands similar to Withaferin A targeting indoleamine 2,3-dioxygenase. *Biosciences Biotechnology Research Asia*.

[20] Kim H. S., Ohno M., Xu B. (2003). 2-Substitution of adenine nucleotide analogues containing a bicyclo[3.1.0]hexane ring system locked in a northern conformation: enhanced potency as P2Y_1_ receptor antagonists. *Journal of Medicinal Chemistry*.

[21] Mustata G., Follis A. V., Hammoudeh D. I. (2009). Discovery of novel myc-max heterodimer disruptors with a three-dimensional pharmacophore model. *Journal of Medicinal Chemistry*.

[22] Eyunni S. K. V. K., Gangapuram M., Redda K. K. (2014). In-vitro antiproliferative activity of new tetrahydroisoquinolines (THIQs) on ishikawa cells and their 3D pharmacophore models. *Letters in Drug Design and Discovery*.

[23] Wang X., Zhang Y. L., Xiang Y. H., Ren Z. Z., Qiao Y. J. (2013). Identification of thrombin inhibitors from salvia miltiorrhiza by pharmacophore based virtual screening and molecular docking. *China Journal of Traditional Chinese Medicine Pharmacy*.

[24] Jiang L., Li Y., Qiao L. (2015). Discovery of potential negative allosteric modulators of mGluR5 from natural products using pharmacophore modeling, molecular docking, and molecular dynamics simulation studies. *Canadian Journal of Chemistry*.

[25] Lipinski C. A. (2004). Lead- and drug-like compounds: the rule-of-five revolution. *Drug Discovery Today: Technologies*.

[26] Huang S.-Y., Grinter S. Z., Zou X. (2010). Scoring functions and their evaluation methods for protein-ligand docking: recent advances and future directions. *Physical Chemistry Chemical Physics*.

[27] Shimada S., Komamura K., Kumagai H., Sakurai H. (2004). Inhibitory activity of shiitake flavor against platelet aggregation. *BioFactors*.

[28] Kim T. H., Ku S.-K., Bae J.-S. (2012). Antithrombotic and profibrinolytic activities of eckol and dieckol. *Journal of Cellular Biochemistry*.

[29] Yong Y. L., Sanghyun L., Jing L. J., Hye S. Y.-C. (2003). Platelet anti-aggregatory effects of coumarins from the roots of Angelica genuflexa and A. gigas. *Archives of Pharmacal Research*.

[30] Li R. S., Li J. L., Xu J. J. (1987). Stimulatory action of *Epimedium sagittatum* (sieb. et zucc.) maxim. on platelet aggregation in rats. *Zhong Yao Tong Bao*.

[31] Waqar M. A., Mahmood Y., Saleem A., Saeed S. A. (2009). An investigation of platelet anti-aggregation activity in indigenous medicinal herbs. *Journal of the Chemical Society of Pakistan*.

[32] Wu T.-S., Tsang Z.-J., Wu P.-L. (2001). New constituents and antiplatelet aggregation and anti-HIV principles of Artemisia capillaris. *Bioorganic & Medicinal Chemistry*.

[33] Wu T.-S., Hsu H.-C., Wu P.-L., Teng C.-M., Wu Y.-C. (1998). Rhinacanthin-Q, a naphthoquinone from *Rhinacanthus nasutus* and its biological activity. *Phytochemistry*.

[34] Liao Y.-R., Leu Y.-L., Chan Y.-Y., Kuo P.-C., Wu T.-S. (2012). Anti-platelet aggregation and vasorelaxing effects of the constituents of the rhizomes of zingiber officinale. *Molecules*.

[35] Gilani A. H., Mehmood M. H., Janbaz K. H., Khan A.-U., Saeed S. A. (2009). Gut modulatory and antiplatelet activities of *Viscum cruciatum*. *Pharmaceutical Biology*.

